# Final grain weight is not limited by the activity of key starch-synthesising enzymes during grain filling in wheat

**DOI:** 10.1093/jxb/ery314

**Published:** 2018-08-25

**Authors:** Brendan Fahy, Hamad Siddiqui, Laure C David, Stephen J Powers, Philippa Borrill, Cristobal Uauy, Alison M Smith

**Affiliations:** 1 John Innes Centre, Norwich Research Park, Norwich, UK; 2 Rothamsted Research, West Common, Harpenden, Hertfordshire, UK

**Keywords:** Field crop, grain development, grain weight, landrace, starch synthesis, wheat, *Triticum aestivum*

## Abstract

Since starch is by far the major component of the mature wheat grain, it has been assumed that variation in the capacity for starch synthesis during grain filling can influence final grain weight. We investigated this assumption by studying a total of 54 wheat genotypes including elite varieties and landraces that were grown in two successive years in fields in the east of England. The weight, water content, sugars, starch, and maximum catalytic activities of two enzymes of starch biosynthesis, ADP-glucose pyrophosphorylase and soluble starch synthase, were measured during grain filling. The relationships between these variables and the weights and starch contents of mature grains were analysed. Final grain weight showed few or no significant correlations with enzyme activities, sugar levels, or starch content during grain filling, or with starch content at maturity. We conclude that neither sugar availability nor enzymatic capacity for starch synthesis during grain filling significantly influenced final grain weight in our field conditions. We suggest that final grain weight may be largely determined by developmental processes prior to grain filling. Starch accumulation then fills the grain to a physical limit set by developmental processes. This conclusion is in accord with those from previous studies in which source or sink strength has been artificially manipulated.

## Teaser

In field conditions that favour high yields, final grain weight of wheat is not limited by sugar availability or activities of key enzymes of starch synthesis during grain filling.

## Introduction

The aim of this work was to establish whether final grain weight is dependent on the capacity for grain filling (the accumulation of storage products) in field-grown wheat (*Triticum aestivum*). Wheat yield per unit land area is a function of the weight of individual grains and the numbers of grains per unit land area (influenced by numbers of ears per plant and number of filled grains per ear). Numbers of grains per unit land area is a complex, weakly inherited trait, but individual grain weight is more stably inherited (Kuchel *et al.*, 2007; Sadras and Slafer, 2012). Although there is frequently a trade-off between individual grain weight and grain number, this relationship may not be due to competition (Acreche and Slafer, 2005; Ferrante *et al.*, 2015). Indeed, it is possible to produce wheat varieties that have both high grain numbers per unit land area and high individual grain weights. Robust quantitative trait loci (QTL) that increase grain weight without reducing grain number have been identified (Simmonds *et al.*, 2014, Griffiths *et al.*, 2015). Understanding how individual grain weight is determined could thus contribute to efforts to increase wheat yields.

Many previous attempts to understand and to increase final grain weight in wheat have focussed on substrate availability and the capacity for starch synthesis during grain filling. Since starch is by far the major component of the mature grain, it has been assumed that variation in the capacity for starch synthesis during grain filling can influence final grain weight. There is some experimental support for this assumption. Manipulation of ADP-glucose pyrophosphorylase (AGPase), the first committed enzyme of starch synthesis, is reported to increase final grain weight under some conditions. Specifically, transgenic wheat plants with elevated AGPase activity during grain filling have been reported to have both more grains and higher yields (Smidansky *et al.*, 2002; Kang *et al.*, 2013*a*), and in one case higher individual seed weight (Kang *et al.*, 2013*a*). Haplotypes of genes encoding both the large and the small subunits of AGPase are also reported to show association with wheat grain weight (Rose *et al.*, 2016; Hou *et al.*, 2017). Indirect evidence for the importance of AGPase comes from experiments in which developing wheat ears were treated with a chemical from which the sugar-signalling molecule trehalose 6-phosphate (T6P) is released by UV light. T6P is believed to increase starch synthesis, putatively through activation of AGPase (Kolbe *et al.*, 2005; Lunn *et al.*, 2006) and elevated expression of genes encoding enzymes of starch synthesis (Zhang *et al.*, 2009; Griffiths *et al.*, 2016). Application of T6P to wheat ears was found to result in larger grains with elevated starch contents on a weight basis (Griffiths *et al.*, 2016). The capacity for starch synthesis was not assessed, but the data are consistent with the idea that T6P produces larger grains by accelerating starch synthesis.

There is also evidence that the second enzyme of starch synthesis, starch synthase (SS), may be important for grain filling. First, SS activities are barely sufficient to support the rate of starch synthesis throughout wheat grain filling, and the rate of starch synthesis and SS activity decline in parallel as grains mature (Hawker and Jenner, 1993; Tomlinson and Denyer, 2003; Zahedi and Jenner, 2003; Yang *et al.*, 2004; Zhang *et al.*, 2010). Other enzymes on the sucrose-to-starch pathway have much higher activities than required to support the flux to starch. Second, the widely observed deleterious effect of temperatures above ~25 °C on wheat grain starch synthesis and hence on grain size and yield is partly attributable to the properties of SS. It is reported to have very low Q_10_ values and to exhibit thermal instability, and elevated temperatures may reduce SS gene transcription (Bhullar and Jenner, 1986; Rijven, 1986; Hawker and Jenner, 1993; Keeling *et al.*, 1993; Denyer *et al.*, 1994; Jenner, 1994; Hurkman *et al.*, 2003; Zahedi and Jenner, 2003; Zahedi *et al.*, 2003). Reduction of SS activity in developing grain by short heat pulses has led to the conclusion that grain filling is controlled predominantly by SS, particularly at high temperatures (Keeling *et al.*, 1993). Overall, these observations suggest that variation in endosperm AGPase and/or SS activity during grain development can affect starch accumulation and hence may affect final grain weight.

Increased supply of sucrose into the developing grain can also influence final grain weight, but the basis and extent of this effect is unclear. Expression of the barley sucrose transporter HvSUT1 in transgenic wheat increases the capacity for sucrose uptake into developing grains and also increases final grain weight, but has minor or variable effects on starch, sucrose, and protein concentrations in the grain (Weichert *et al.*, 2010, 2017; Saalbach *et al.*, 2014). Elevated grain weight in this case may be due to increased endosperm cell numbers rather than increased storage-product synthesis (Weichert *et al.*, 2017). The idea that increased supply of assimilates generally increases grain growth is contradicted by numerous physiological experiments in which the relationship between assimilate supply (i.e. leaves, the ‘source’) and the capacity of the grain to use assimilates for growth (the ‘sink’) is manipulated, either by removal of grains or by removal or shading of leaves (e.g. Reynolds *et al.*, 2009; Serrago *et al.*, 2013, and discussions therein).

Despite indications that substrate availability and starch synthesis capacity during grain filling can affect final grain weight, the general relationship between these variables has not been systematically investigated. Most studies of starch synthesis capacity have examined relatively few genotypes (Kang *et al.*, 2010; Wang *et al.*, 2014), and recent studies have examined transcript levels rather than enzyme activities (Kang *et al.*, 2013*b*; Yu *et al.*, 2016). Extrapolation from transcript levels to enzymatic capacity is problematic: the enzymes are encoded by multiple genes (Stamova *et al.*, 2009) and changes in transcript levels do not necessarily result in changes in protein contents or activities (Fernie and Stitt, 2012).

In order to establish the extent to which final grain weight is influenced by substrate availability and the capacity for starch synthesis during grain filling, we measured the maximum catalytic activity (an estimate of the total capacity of the tissue for a particular enzymatic reaction) of AGPase and SS, and the levels of carbohydrate substrates for starch synthesis during grain filling in a large panel of diverse genotypes of hexaploid wheat, and examined the relationship between these values, the accumulation of starch, and the weight of individual grains at maturity. To provide insights relevant to the wheat crop, samples were taken from field-grown plants, in two successive years. Plants were grown in Norfolk, UK, an area of high wheat yields with a moderate climate.

## Materials and methods

### Growth and sampling

Elite cultivars of wheat (*Triticum aestivum*) were from the Earliness & Resilience for Yield in a Changed Climate (ERYCC) panel generated with funding from the Department for Environment, Food and Rural Affairs (UK Government) and the UK Home-Grown Cereals Authority (Project LK0992: Clarke *et al.*, 2012). Landraces were from the A.E. Watkins landrace cultivar collection (Wingen *et al.*, 2014), a global sample of landraces and early varieties that contains useful variation for agronomic traits (Miller *et al.*, 2001).

Winter wheat was grown in plots (one per genotype) of 1.5 × 1.5 m at Church Farm, Bawburgh, Norfolk, UK, and Morley Farm, Wymondham, Norfolk, UK (locations 10 km apart). In 2013, elite cultivars were grown at Morley and landraces at Bawburgh. In 2014, a subset of 21 elite cultivars and landraces was grown on 1.5 × 1.5 m plots at Bawburgh.

Individual primary ears from plants inside the plot were tagged on the day on which they underwent anthesis. At time-points during development, ears were harvested from six plants per genotype, sealed in plastic bags and frozen in liquid nitrogen, then stored on dry ice for transport to a –80 °C freezer. Samples of grains for enzyme and metabolite assays were dissected into pre-weighed tubes, on dry ice. Each sample contained 10 grains from the outer (most proximal to the rachis) florets (positions F1 and F2) of spikelets in the central region (from 25–75% of the base-to-tip distance) of a single ear. Following weighing, sample tubes were stored at –80 °C.

Grain was harvested after whole-plant senescence (complete yellowing of leaves, peduncle, and spike). This occurred after physiological grain maturity for all samples in both years. Mature grain samples were harvested and conditioned over at least 4 weeks in a common environment to ensure a common moisture content and comparability regardless of the exact date at which physiological maturity was reached. In 2013, mature grain was bulked for each genotype, then three subsamples were taken for weight and starch assays. In 2014, mature grain was harvested from the centre of a single primary ear each of six randomly selected plants per genotype.

From anthesis to harvest in both 2013 and 2014 the average maximum daily temperature was 22 °C. The temperature was over 25 °C on six days in 2013 and on three days in 2014. The highest temperatures were 29 °C in 2013 and 27 °C in 2014 (data from Buxton Weather Station, ~8 km from Bawburgh, by kind permission of Charles Briscoe; www.buxton-weather.co.uk/; accessed 05/09/18).

### Grain weight

For developing grains, fresh weight was measured and then samples were dried at 60 °C for 72 h and reweighed to obtain dry weight. The two values were used to calculate water content. Final grain weight was measured using a Marvin seed analyser (www.gta-sensorik.com; accessed 05/09/18).

### Enzyme assays

Samples of ~175 mg frozen grain were homogenized at 28 vibrations s^–1^ and 4 °C for 2 min on a Retsch ball mill (www.retsch.com; accessed 05/09/18) in a tube containing a 7-mm steel ball and ~0.5 ml of 100 mM MOPS/NaOH, pH 7.2, 5 mM MgCl_2,_ 5% v/v glycerol, 5 mM DTT, 10 mg ml^–1^ bovine serum albumin, 1% (w/v) polyvinylpolypyrrolidone, to give an extract concentration of 350 mg tissue ml^–1^. Insoluble material was pelleted by centrifugation and the supernatant used for assays.

Assay components were optimised to give maximum activities on extracts of developing grains. We checked that reaction rates were constant over time, and dependent on appropriate substrates and on extract concentration.

AGPase was assayed at 25 °C and 340 nm in a microtitre plate reader. Assays were conducted in duplicate and contained, in 200 µl, 100 mM MOPS/NaOH, 2.5 mM MgCl_2_, 2 mM ADP-glucose, 1 unit glucose 6-phosphate dehydrogenase (from *Leuconostoc mesenteroides*; www.roche.co.uk; accessed 05/09/18), 1 unit phosphoglucomutase (www.sigmaaldrich.com ; accessed 05/09/18, P3397), 1.5 mM Na pyrophosphate (used to initiate the assay), and 40 µl extract.

Starch synthase was assayed at 25 °C by the ADP^14^C glucose method of Jenner *et al.* (1994). Assays were conducted in duplicate and contained, in 100 µl, 100 mM glycylglycine, pH 8.2, 25 mM Na acetate, 10 mM DTT, 0.5 mg potato amylopectin, 2 mM ADP-glucose, and 10 µl extract.

### Metabolite assays

Samples of ~350 mg frozen grain were homogenized on a Retsch ball mill (settings as for enzyme assays, above) in 0.5 ml of 0.77 M perchloric acid. After 45 min at 0 °C, debris was pelleted by centrifugation and the supernatant removed. The pellet was washed by resuspension in water and centrifugation, and the washings added to the supernatant. The combined supernatant was neutralized with 2 M KOH, 0.4 M MES, 0.4 M KCl and used for sugar assays following removal of the precipitate by centrifugation. The pellet was washed three times by resuspension in 80% v/v ethanol, and used for starch assays.

Sugars and starch were assayed enzymatically according to Critchley *et al.* (2001) and Delatte *et al.* (2005), using a microtitre plate reader.

### Statistics and data displays

For ease of interpretation, data in most figures are shown without error bars and designation of individual genotypes: full datasets with measures of variance are provided in Supplementary Table S1 at *JXB* online. Pearson’s correlation coefficient, *r*, was calculated between pairs of variables of interest using the means of data per cultivar to ensure independent observations given that the field trials used single plots. Correlations were tested for statistical significance using the *F*-test and a significance level of *P*<0.05 was used. The multivariate techniques Principal Coordinates (PCo) analysis and cluster analysis were used to assess the similarity between cultivars. Euclidean distance measures of similarity were used to reduce the 21 (genotypes) × 68 responses (data points: Supplementary Table S2) data matrix from 2014 and the 21 × 44 (elite cultivars) and 36 × 44 (landraces) data matrices for 2013 to 21 × 21 and 36 × 36 similarity matrices, to which PCo analysis was applied (Supplementary Table S3).

The PCo allowed a reduced dimensional representation of the similarity matrix, thus allowing inspection of the distribution and separation of the cultivars in two-dimensional plots. The cluster analysis used the group average method of clustering and it provided a dendrogram that allowed identification of potential groups of cultivars at particular levels of similarity. The Genstat (18th edition, 2015, © VSN International Ltd, Hemel Hempstead, UK) statistical package was used for all analyses.

## Results

### Sampling strategy and year-on-year variation

To assess the extent of variation for maximum catalytic activities of AGPase and SS and sugar and starch contents between genotypes, and to look for correlations between these variables and final grain weight, we harvested field-grown developing and mature grain from 18 UK elite cultivars and 36 landraces in 2013, and from a subset of these lines (representing the full spread of final grain weights) in 2014. Detailed comparisons between landraces and the elite cultivars in 2013, and between 2013 and 2014 datasets, are precluded by differences between these three trials in field locations and in mature grain sampling strategies (see Methods). The three datasets (in full in Supplementary Table S1) are largely considered separately under the headings below. Nonetheless, between-year comparisons of selected variables can provide insights into the environmental plasticity of grain maturation across the panel of landraces and elite cultivars.

**Fig. 1.**
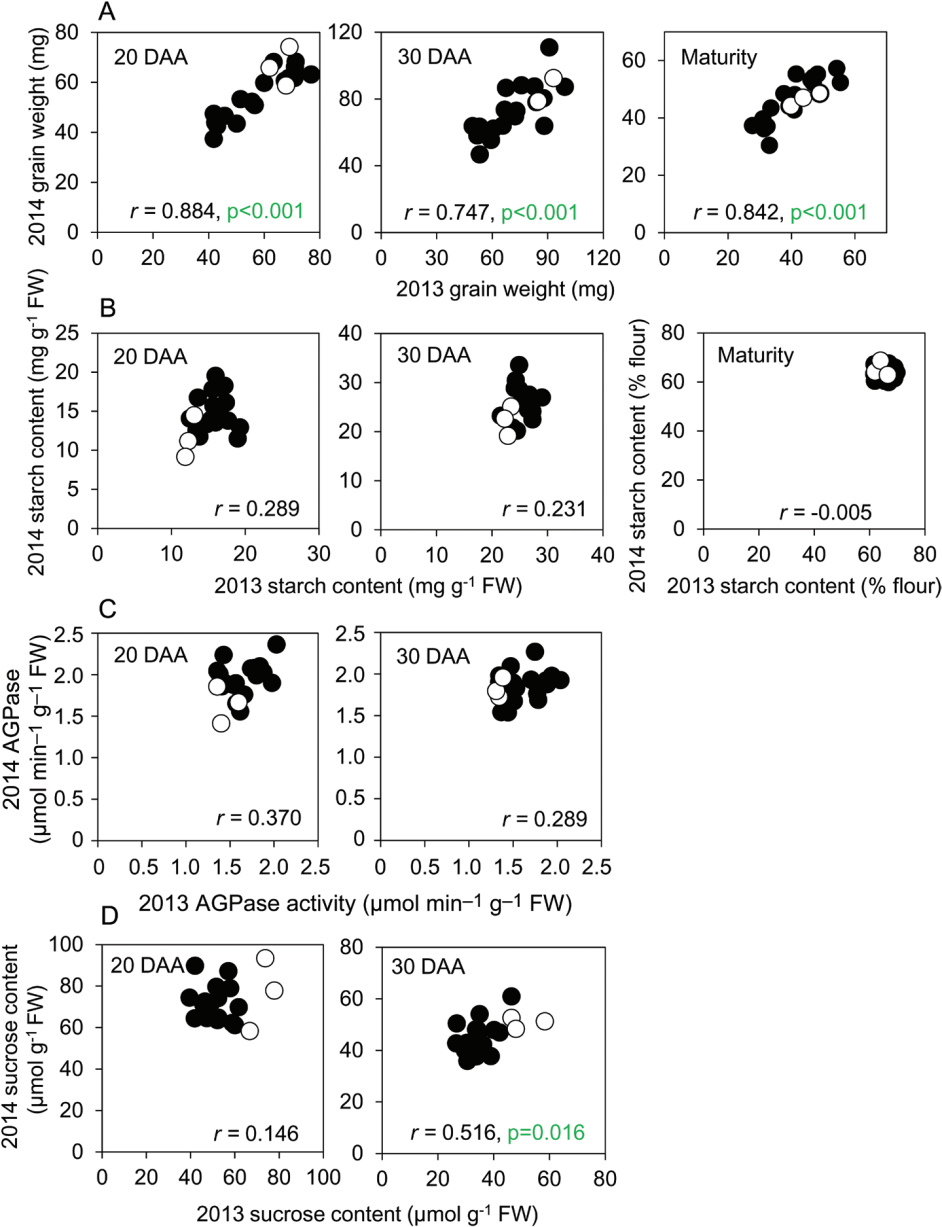
Comparisons of genotypes between 2013 and 2014. For each genotype grown in both years, 2013 values are plotted against 2014 values. Black symbols are landraces; open symbols are elite cultivars. The graphs show sampling time as days after anthesis (DAA), Pearson’s correlation (*r*), with *n*=21, *P*-values (*F*-test) where *P*<0.05 (i.e. where no value is shown, *P*>0.05). (A) Grain fresh weight, (B) starch content, (C) AGPase activity, and (D) sucrose content. Full datasets including the genotype designations and measures of variance are provided in Supplementary Table S1, and Supplementary Fig. S1 shows final grain weights plotted as bar charts.

When the whole panel of genotypes was considered together, final grain weights for the genotypes in 2013 were strongly correlated with their final grain weights in 2014. This suggests a relatively small influence of environment on grain weight between these two growing seasons (Fig. 1A; for genotype designations see Supplementary Fig. S1). The starch contents of mature grains varied little with either genotype or year, and there was no significant correlation between starch contents of developing grains in 2013 and 2014 [Fig. 1B; *P*=0.202 for grains at 20 d after anthesis (DAA), *P*=0.312 for 30 DAA]. Maximum catalytic activities of AGPase and sucrose content on a fresh-weight basis in developing grains also showed little correlation between 2013 and 2014 (Fig. 1C, D). These data suggest that—across the two years of our experiment–final grain weight was under strong genetic control, while starch content, enzyme activity, and sucrose content were more susceptible to environmental modulation.

### Fresh- and dry-weight gain during grain development

**Fig. 2.**
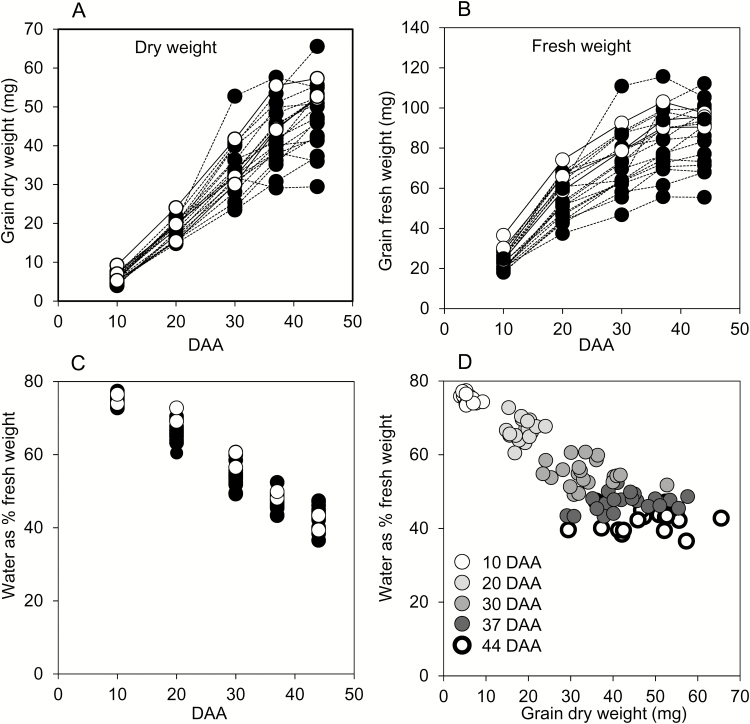
Fresh- and dry-weight gain through grain development for genotypes grown in 2014. Each point represents the value from a single genotype, and is the mean of measurements made on six samples, each from a different plant, consisting of grain from the central portion of a single ear. Full datasets including the genotype designations and measures of variance are provided in Supplementary Table S1. Black symbols and dashed lines are landraces; white symbols and solid lines are elite cultivars. DAA, days after anthesis. (A) Grain DW, (B) grain FW, and (C) water content through development. Lines are omitted for clarity in (C). (D) Water content plotted against grain DW at the DAA indicated.

Most studies of enzyme activities and metabolite contents in developing grain express data on a fresh-weight or per-grain basis. It is not possible to measure the dry weight of samples used for metabolic measurements. However, measurements of other variables in developing grain frequently use a dry-weight basis. To allow meaningful comparisons with other studies, we measured both FW and DW increases during grain development in 2014 (Fig. 2A, B). In general, DW increased rather linearly up to 37 DAA, then more slowly between 37 and 44 DAA. FW doubled between 10 and 20 DAA, then slowed progressively up to 44 DAA. For nearly half of the genotypes, FW reached its maximum by 37 DAA. Water content (as percent of FW) decreased from 10 to 44 DAA, and was similar across genotypes (Fig. 2C). Small grains had the same progressive loss of water through development as large grains (Fig. 2D). Because genotype and grain size had little impact on water content, expression of data on either a FW or a DW basis is expected to yield qualitatively similar conclusions.

When measurements are made on a per-grain basis, differences between genotypes and developmental stages are strongly influenced by differences in grain size, potentially obscuring other interesting trends. Expression on a weight basis provides more readily interpretable information about differences in substrate availability and the capacity for starch synthesis. To enable broad comparisons, we present enzyme activity and metabolite data on both a FW and a grain basis. Most of the measurements in the main figures are on a FW basis; measurements on a grain basis are largely in the Supplementary data.

### Changes in maximum catalytic activities of AGPase and SS during development

**Fig. 3.**
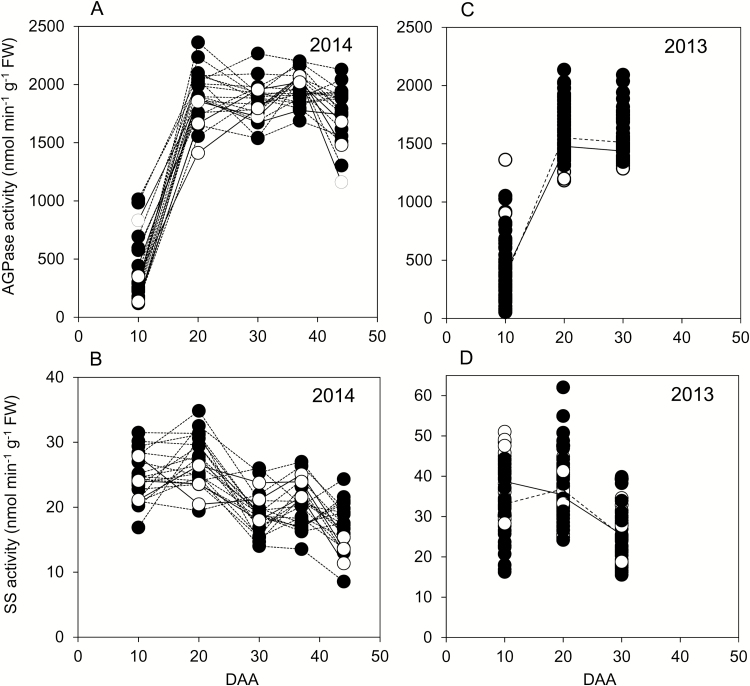
Enzyme activities through grain development. Values represented by each point are as described in Fig. 2. Full datasets including the genotype designations and measures of variance are provided in Supplementary Table S1. DAA, days after anthesis. (A) AGPase and (B) starch synthase (SS) activities for genotypes grown in 2014. Black symbols and dashed lines are landraces; white symbols and solid lines are elite cultivars. ((C) AGPase and (D) SS activities for genotypes grown in 2013. Symbols are as for (A, B); solid lines join mean values for elite cultivars; dashed lines join mean values for landraces.

For genotypes grown in 2014, AGPase activity rose strongly on a FW basis between 10 and 20 DAA (Fig. 3A). Activity remained high between 20 and 44 DAA, although it declined somewhat between 37 and 44 DAA in more than half of the genotypes. SS activity followed a different pattern (Fig. 3B). In most genotypes activity was highest at 10 or 20 DAA, followed by a decline. Activity was lower at 37 and at 44 DAA than at 20 DAA for all but two genotypes. The trends in enzyme activities between 10 and 30 DAA in 2014 were also present in the larger set of genotypes grown in 2013. AGPase activity rose strongly from 10 to 20 DAA, then levelled off (Fig. 3C). For both elite cultivars and landraces, mean SS activity was lower at 30 DAA than at 10 or 20 DAA (Fig. 3D).

### Changes in starch and sugar levels during development

**Fig. 4.**
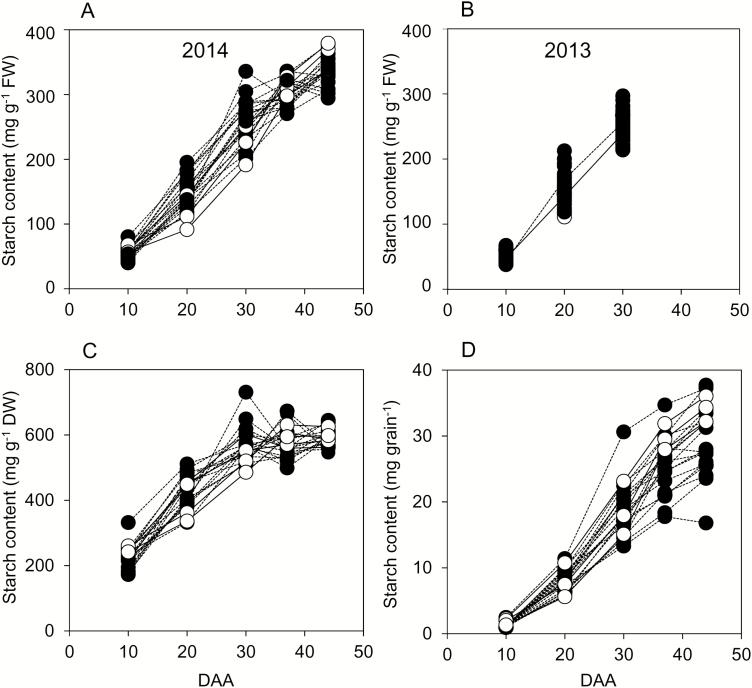
Starch contents through grain development. Values represented by each point are as described in Fig. 2. Full datasets including the genotype designations and measures of variance are provided in Supplementary Table S1. DAA, days after anthesis. Black symbols and dashed lines are landraces; white symbols and solid lines are elite cultivars. (A) Starch content on a FW basis for genotypes grown in 2014. (B) Starch content on a FW basis for genotypes grown in 2013 (the solid line joins mean values for elite cultivars; the dashed line joins mean values for landraces). (C) Starch content on a DW basis for genotypes grown in 2014. (D), Starch content on a per-grain basis for genotypes grown in 2014.

For essentially all genotypes in both years, starch accumulated rapidly from 10 to 30 DAA (Fig. 4). Rapid accumulation continued up to 44 DAA in some genotypes, but for most the rate of accumulation on a FW basis slowed between 30 and 44 DAA. On a DW basis, there was little change in starch content between 37 and 44 DAA (Fig. 4C); thus the increase in starch content on a FW basis over this period was in part due to water loss from the grain. However, starch content per grain continued to increase between 37 and 44 DAA for most genotypes (Fig. 4D), and hence starch synthesis must have continued over this period. These data indicate that starch synthesis made a major contribution to dry weight gain up to 37 DAA but after that, although it continued, its contribution to dry weight gain was lower than at earlier stages of growth. There were generally only small differences in starch content between 44 DAA and maturity (Supplementary Fig. S2). On a DW basis, starch content averaged across all genotypes was 7% higher at maturity than at 44 DAA. Thus, for most genotypes starch accumulation was largely complete by 44 DAA.

**Fig. 5.**
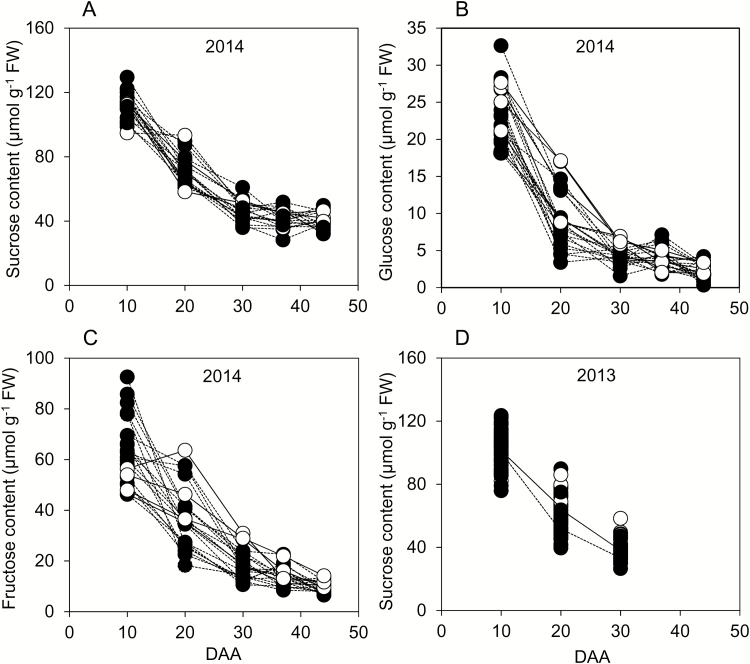
Sugar contents through grain development. Values represented by each point are as described in Fig. 2. Full datasets including the genotype designations and measures of variance are provided in Supplementary Table S1. DAA, days after anthesis. Black symbols and dashed lines are landraces; white symbols and solid lines are elite cultivars. (A) Sucrose, (B) glucose, and (C) fructose contents on a FW basis for genotypes grown in 2014. (D) Sucrose content on a FW basis for genotypes grown in 2013 (the solid line joins mean values for elite cultivars; the dashed line joins mean values for landraces).

Sucrose levels declined strongly from 10 to 30 DAA, then fell much more slowly or levelled off from 30 to 44 DAA (Fig. 5A, D). Glucose levels were 4- to 5-fold lower than sucrose levels at 10 DAA and fell more steeply through development, so that they were 10-fold lower than sucrose levels at 44 DAA (Fig. 5B). Fructose levels were intermediate between those of sucrose and glucose: levels fell through development, and at 44 DAA were less than one third of sucrose values (Fig. 5C). The ratio of sucrose to hexoses increased through development: hexose and sucrose contents were similar at 10 DAA but hexose content was one-third of sucrose content at 44 DAA.

### Correlations between final grain weight, enzyme activities, and metabolite levels

**Fig. 6.**
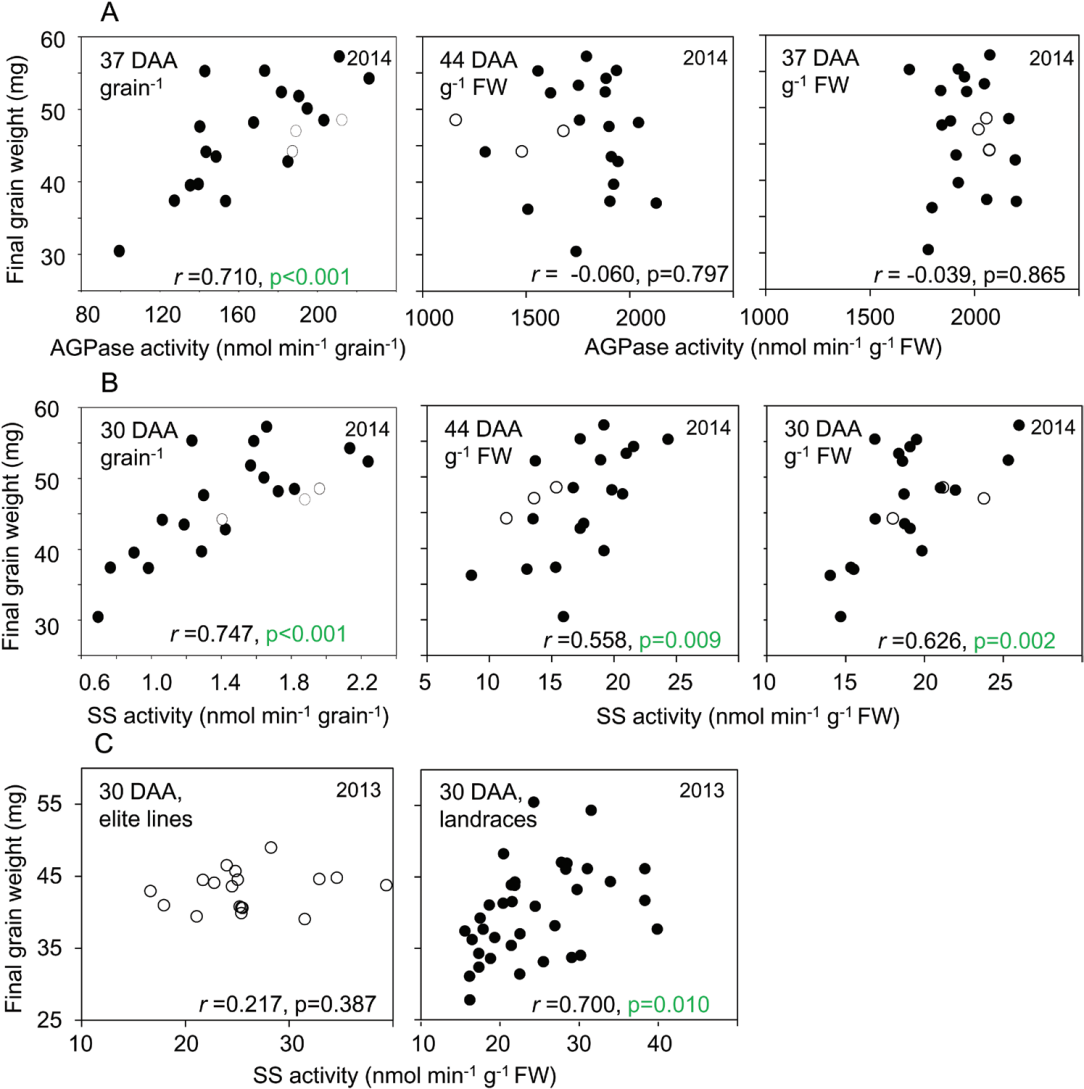
Relationships between final grain weight and enzyme activities on a fresh-weight and a per-grain basis. Representative examples of the data are plotted; further plots are provided in Supplementary Fig. S3. Values are means taken from Supplementary Table S1, which also provides full datasets including the genotype designations and measures of variance. The same values are also displayed in different contexts in Fig. 3 (enzyme activities) and Supplementary Figs. S1 and S3 (mature grain weight, and additional relationships between final grain weight and enzyme activities, respectively). Black symbols are landraces; white symbols are elite cultivars. Pearson’s correlation coefficient (*r*) is indicated, with *n*=1; *P*-values (*F*-test) are indicated where *P*<0.05 (i.e. where no value is shown, *P*>0.05) (see Supplementary Table S2). DAA, days after anthesis. (A) Correlations in 2014 between AGPase activity and final grain weight, on a FW basis at 44 DAA and 37 DAA (left and centre, respectively) and on a per-grain basis at 37 DAA (right). (B) As for (A) but for starch synthase (SS) activity at 44 DAA and 30 DAA. (C) Correlations between SS activity at 30 DAA for elite cultivars (left) and landraces (right) grown in 2013.

**Fig. 7.**
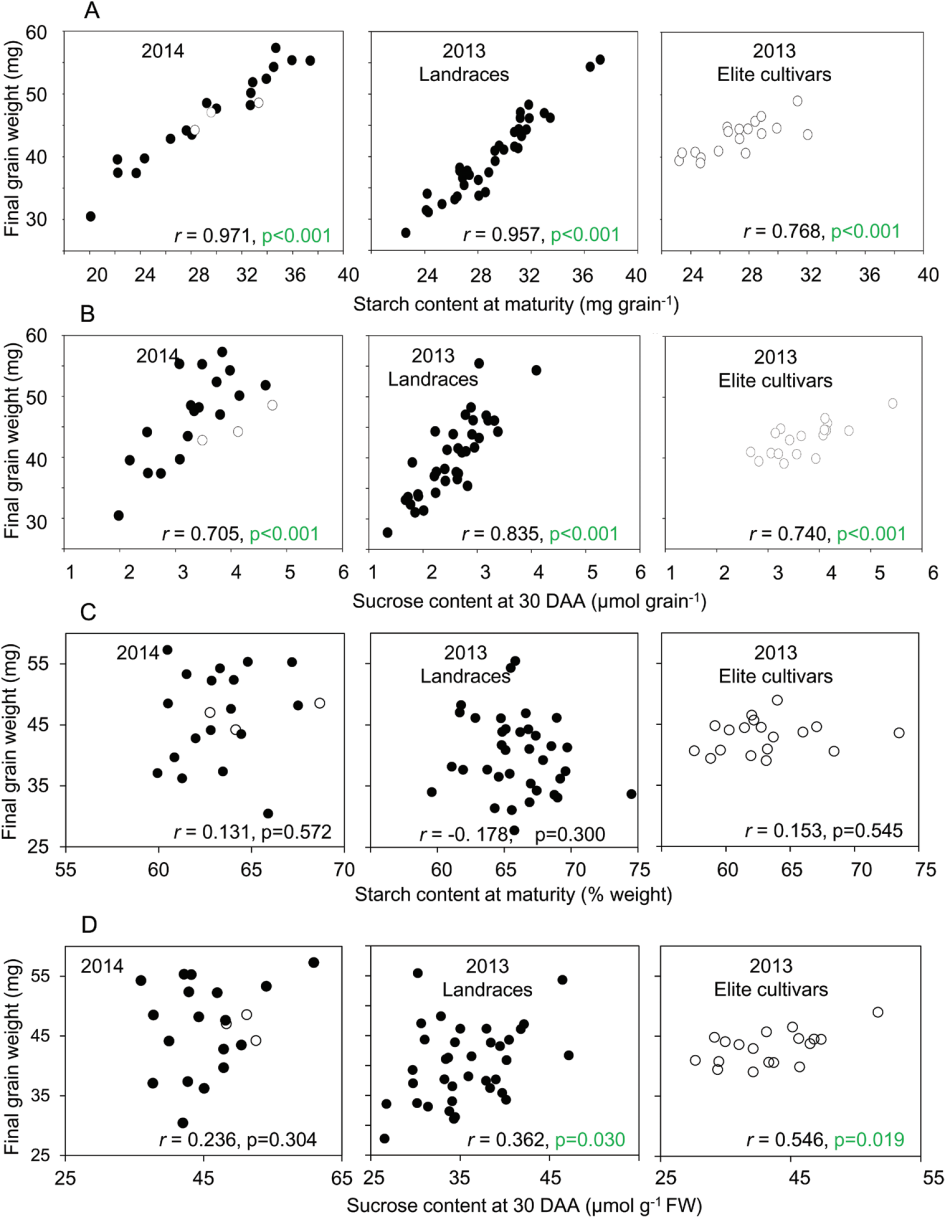
Relationships between final grain weight and carbohydrate contents. A subset of the data is plotted. Values are means taken from Supplementary Table S1, which also provides full datasets including the genotype designations and measures of variance. The same values are also displayed in different contexts in Fig. 5 (sucrose content), and Supplementary Figs S1, S2 (final grain weight and starch content at maturity, respectively). Black symbols are landraces; white symbols are elite cultivars. Pearson’s correlation coefficient (*r*) is indicated, with *n*=21; *P*-values (*F*-test) are in indicated where *P*<0.05 (i.e. where no value is shown, *P*>0.05) (see Supplementary Table S2). (A, C) Correlations between final grain weight and starch content expressed (A) on a per-grain basis and (C) on a weight basis for the mixture of genotypes grown in 2014, landraces grown in 2013, and elite cultivars grown in 2013. (B, D) Correlations between final grain weight and sucrose content expressed (B) on a per-grain basis and (D) on a weight basis for the same lines as in (A, B).

Data for grain fresh weights, enzyme activities, and metabolite levels during development and final grain weights were used to construct correlation matrices for the landraces and the elite cultivars grown at separate sites in 2013, and for the mixture of lines grown at a single site in 2014. As expected, strong, positive correlations of final grain weight with enzyme activities and metabolite levels during grain development were observed when values were expressed on a per-grain basis (Figs 6, 7, Supplementary Fig. S3). In 2014, final grain weight was correlated with enzyme activities during development (Fig. 6; Supplementary Table S2). In 2013, final grain weight was correlated with enzyme activities at 20 DAA and 30 DAA among landraces, but not among elite cultivars (Supplementary Table S2). Final grain weight was also strongly correlated with starch content per grain at maturity (Fig. 7A) and at points during development (Supplementary Table S2), and with sucrose content per grain at points during development (Fig. 7B). By contrast, final grain weight was not correlated, or only weakly correlated, with hexose contents per grain (Supplementary Table S2).

Correlations between grain weight and enzyme activities and metabolite levels were far fewer and weaker when these variables were expressed on a FW rather than a per-grain basis (Figs 6, 7, Supplementary Fig. S3). There were no significant correlations between final grain weight and AGPase activity on a FW basis (Fig. 6A, Supplementary Table S2). However, there were some significant (*P*<0.05, *F*-test) correlations between final grain weight and SS activity on a FW basis in both years: for activity at 10 DAA and 30 DAA in landraces in 2013, and for activity at 30 DAA and 44 DAA in 2014 (Fig. 6B, C, Supplementary Table S2).

There were no significant correlations between final grain weight and either starch at maturity (Fig. 7C) or starch or hexose during development on a FW basis (Supplementary Table S2). In 2013, but not 2014, there were some significant (*P*<0.05, *F*-test) correlations of final grain weight with sucrose content at 30 DAA on a FW basis (Fig. 7D, Supplementary Table S2).

### Visualisation of similarities between genotypes

To provide information about degrees of similarity between genotypes, PCo analysis was applied to similarity matrices constructed for the landraces and the elite cultivars grown at separate sites in 2013, and for the mixture of lines grown at a single site in 2014 (see Methods; Supplementary Table S3).

In 2013, the first three PCo accounted for 62% and 65% of the variation in the distance matrix for the elite cultivars and landraces, respectively. For the mixture of landraces and elite cultivars grown in 2014, the first three PCo accounted for 56% of the variation in the distance matrix. Inspection of plots of pairs of PCo (Supplementary Fig. S4) revealed genotypes that were outliers from the clouds of points: the nature and potential significance of these observations are discussed in Supplementary Text S1.

**Fig. 8.**
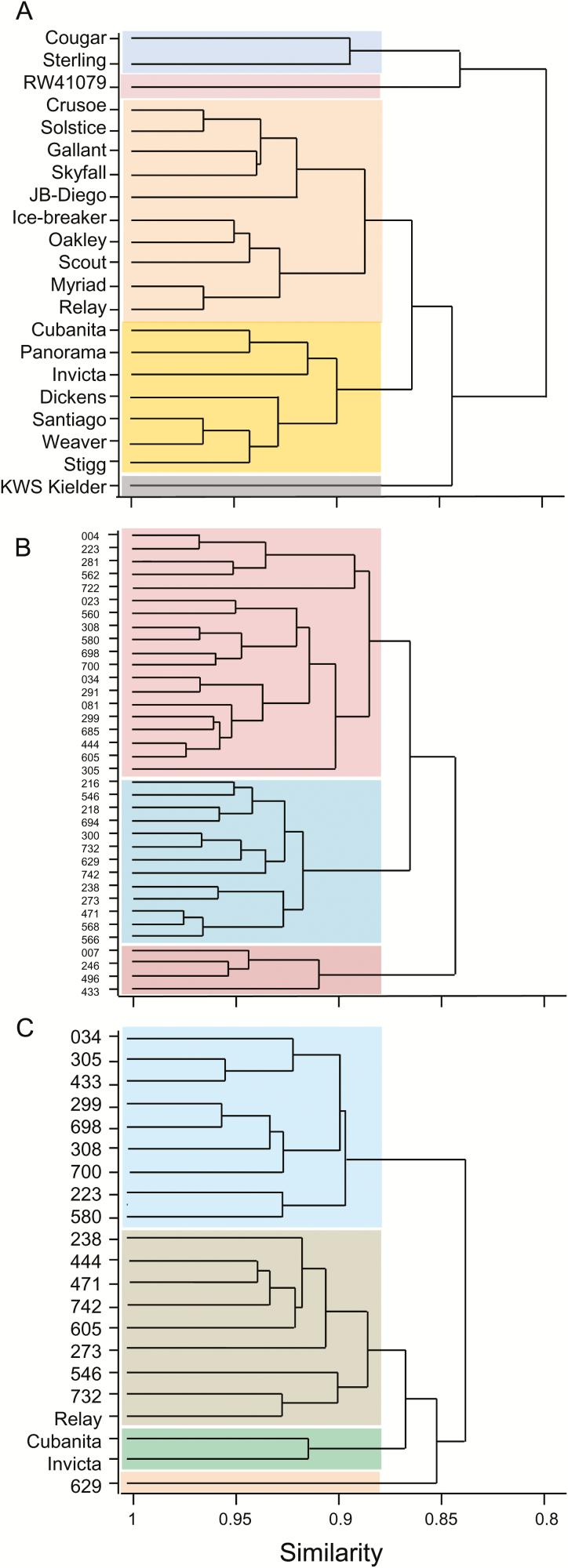
Dendrograms illustrating similarities between genotypes. The dendrograms are derived from similarity matrices constructed for the 21 genotypes used in 2014 and the 21 elite cultivars and 36 landraces used in 2013. Euclidean distance measures of similarity were used to reduce the 21 × 68 data matrix from 2014 and the 21 × 44 (elite cultivars) and 36 × 44 (landraces) data matrices for 2013 to 21 × 21 and 36 × 36 similarity matrices. The coloured blocks distinguish genotypes clustered with a similarity value <0.88. For landraces, the nomenclature is abbreviated by removal of the initial 1190 common to all genotypes (see Supplementary Table S1). (A) Elite cultivars grown in 2013, (B) landraces grown in 2013, and (C) the mixture of genotypes grown in 2014.

Cluster analyses based on the 2013 similarity matrices (Fig. 8A, B) showed less similarity within the elite cultivars than within the landraces. For a similarity score of 0.88, for example, there were five clusters of elite cultivars but only three clusters of landraces. We checked the clusters for elite cultivars against their pedigrees, their grain-quality classifications (http://www.nabim.org.uk/wheat-varieties), the year of their release, and the number of years in which they were on the UK Recommended List for winter wheats. None of these measures provided an explanation for the clusters derived from the similarity matrix (Supplementary Table S4). A cluster analysis based on the mixture of landraces and elite cultivars grown in 2014 also showed low similarity between elite cultivars: at a similarity score of 0.88, ‘Relay’ was in a different cluster from the other two elite cultivars.

## Discussion

Below, we use our results to evaluate the extent to which final grain weight was determined by the capacity for starch synthesis and/or the supply of assimilate during the main period of grain filling (from 10 DAA onwards). Our conclusions are subject to the limitations of our experimental set-up. The technical demands of assaying enzymes and metabolites on numerous samples meant that it was not possible to replicate the genotypes in independent plots, as is usual practice for field experimentation. Instead, we repeated a large part of the experiment in two growing seasons. Our conclusions are based on consideration of these two, independent sets of analyses.

In general, our results indicated that final grain weight was not strongly influenced by the availability of sugars or the activities of AGPase or starch synthase in the genotypes and under the environmental conditions we studied. We first discuss the roles of sugar availability, starch synthetic capacity, and starch accumulation in determining final grain weight, and compare our data in these individual respects with the outcomes of previous studies. We then evaluate the idea that developmental processes rather than grain filling determine final grain weight, and discuss whether this is likely to be the case for genotypes and environments different from those in our experiments.

### Final grain weight is not dependent on levels of sugars in the grain from 10 DAA onwards

Our data revealed no consistent, positive correlations between final grain weight and levels of sucrose, glucose, and fructose in the grain on a weight basis from 10 DAA onwards (Fig. 7, Supplementary Table S2). In broad terms, these findings imply that the availability of substrate for starch synthesis in the developing grain did not determine final grain weight, and hence that attempts to increase the availability of sugars in the grain during the main period of grain filling are relatively unlikely to result in heavier grains in high-yielding environments.

Other studies have also concluded that starch synthesis during grain filling is not usually limited by the availability of sucrose. For example, manipulation of sucrose supply to grains by the removal of leaves or by provision of exogenous sucrose to detached, developing ears has no major effects on dry weight gain, implying that the supply of sucrose is not limiting for grain growth (Jenner and Rathjen, 1972; Jenner *et al.*, 1991).

Hexoses fell to low levels during the first 30 DAA (Fig. 5). Glucose supply is essential for normal grain development in other cereals (Cheng *et al.*, 1996; Wang *et al.*, 2008; Sosso *et al.*, 2015; Doll *et al.*, 2017), and this may also be the case in wheat. Glucose seems to be important for events early in development rather than for starch synthesis. Reduced import into young grains profoundly affects development of transfer cells in the basal endosperm transfer layer in maize, suggesting a specific, developmental signalling function (Sosso *et al.*, 2015).

### Final grain weight is not strongly dependent on AGPase or starch synthase activity from 10 DAA onwards

We found no significant correlations between final grain weight and AGPase activity on a weight basis from 10 DAA onwards (Fig. 4, Supplementary Table S2). The maximum catalytic activity of AGPase between 20 and 40 DAA (1.5–2 µmol min^–1^ g^–1^ FW; Fig. 3) was much greater than the estimated rate of starch synthesis (maximum 0.04 µmol glucose equivalents min^–1^ g^–1^ FW: see Methods). It is thus unlikely that grain filling and hence final grain weight were limited by AGPase activity during the main part of grain filling.

These results are at odds with the widespread expectation that high AGPase activity in the endosperm leads to high starch content and hence high grain weight (discussed in Tuncel and Okita, 2013; Cakir *et al.*, 2015), and with previous research that supports this expectation. For example, transgenic wheat with elevated AGPase activity in the grain has higher final grain weight than untransformed controls (Kang *et al.*, 2013*a*), and polymorphisms in genes encoding the subunits of AGPase have been associated with variation in final grain weight in two different panels of wheat genotypes (Rose *et al.*, 2016; Hou *et al.*, 2017). We suggest that these effects of AGPase variation on grain weight may not arise during grain filling, but may instead reflect a crucial role for the enzyme in events around the time of anthesis that influence final grain weight via the extent of expansion of enclosing maternal tissues or patterns of endosperm cellularisation. Support for this suggestion is as follows.

There is good evidence that AGPase activity around anthesis can influence numbers of grains that set, and/or very early developmental processes in the grain. Expression of an inhibition-insensitive form of endosperm AGPase in maize increases kernel yield under heat stress: Hannah *et al.* (2012) found that this effect was associated with increased numbers of mature kernels. As well as expression during kernel filling, the transgene was expressed in reproductive tissues around the time of fertilisation. This expression increased the chance that a given ovary would develop into a kernel under heat stress. Genes encoding the main endosperm AGPase of wheat are also expressed in the developing spike prior to fertilization (small subunit genes 7AS_1B2A8C929, 7BS_4FBE4B00A, 7DS_02539EB3B; large subunit genes 1AL_A1B2A8EB0, 1BL_190920E1E, 1DL_844FE40E6; http://www.wheat-expression.com/;Borrill *et al.*, 2016; see also Chrimes *et al.*, 2005), and there is an association between haplotypes for one of these AGPase genes and the numbers of grains per spike (Rose *et al.*, 2016). It has been suggested that reproductive structures are susceptible to starvation-induced abortion around the time of fertilisation, and that starch reserves help to prevent this problem (see McLaughlin and Boyer, 2004). This idea may explain why modulation of AGPase activity in reproductive structures influences grain numbers rather than final grain size.

In contrast to our findings for AGPase, there were some significant correlations between final grain weight and SS activity on a weight basis (Fig. 6). However, SS activity was not correlated with starch content on a weight basis (Supplementary Table S2). It is thus unlikely that SS activity influenced grain weight by determining the amount of starch per unit weight. The mechanistic basis of the relationship between SS activity and grain weight remains unclear.

As described in the Introduction, SS activity is strongly limiting for starch synthesis in wheat grains at temperatures at the upper end of the physiological range. Surprisingly, the molecular basis of this potentially important phenomenon is not known. QTL and association studies have not found robust links between SS haplotypes and either starch content or weight of mature grains, but these approaches are compromised by the complexity of the SS family. Four different SS isoforms contribute to synthesis of the major, amylopectin component of starch, and there is likely to be both synergy and a high degree of redundancy between these forms (Tetlow *et al.*, 2008). A fifth SS, granule-bound starch synthase I (GBSSI) is exclusively responsible for the synthesis of the amylose component of starch, but there is good genetic evidence that it is not important for starch content or weight of mature grains (e.g. Ahuja *et al.*, 2013; Hucl and Ramachandran, 2015; Yamamori and Yasui, 2016).

It remains possible that other steps in starch synthesis in wheat endosperm are important in determining final grain weight. The possibility that control is exerted at the point of the entry of ADP-glucose into the amyloplast via the transporter Brittle1 (Bt1), was investigated by over-expression of *Bt1* in rice endosperm (Cakir *et al.*, 2016). Capacity for ADP-glucose transport into amyloplasts was increased in the transgenic lines but there was no effect on grain weight, indicating that this step exercised little control over starch synthesis under normal circumstances. It is also possible that although variation in activities of individual enzymes have little effect on final grain weight, a simultaneous change in the same direction of most or all of the enzymes on the sucrose-to-starch pathway could have a significant effect (see Fell, 1998).

Our conclusion that that final grain weight was not limited by the maximum catalytic activities of AGPase or starch synthase or by substrate availability during grain filling is seemingly at odds with the demonstration that application of T6P analogues during grain filling increases starch content and final grain size (Griffiths *et al.*, 2016). In Arabidopsis leaves, applications of the same analogues increase the rate of starch synthesis (Griffiths *et al.*, 2016), so it is reasonable to propose that increased starch synthesis may account for greater grain size in T6P-treated wheat ears. We suggest that T6P may modulate multiple aspects of grain metabolism, rather than acting on a single component of the starch biosynthetic machinery. In Arabidopsis seedlings in liquid culture, application of T6P analogues altered the transcript levels for multiple genes under the control of the energy-sensing protein kinase SnRK1 as well as for several enzymes of starch metabolism (Griffiths *et al.*, 2016).

### Final grain weight is independent of starch content on a weight basis

Our data indicated that final grain weight was not strongly related to starch accumulation on a weight basis during grain filling (Fig. 7). If starch accumulation determines grain weight, we would expect large grains to have a higher percentage of starch than small grains. This was not the case: at maturity, there was much more variation between genotypes in grain weight than in starch content on a weight basis. In other words, starch made up a similar percentage of grain weight in all genotypes, regardless of final grain weight. There were no significant correlations between these two variables (Supplementary Table S2). There was also little correspondence between final grain weight and the pattern of accumulation of starch during the main period of grain filling. Although the overall contribution of starch accumulation to the increase in grain dry weight fell from 30 DAA onwards (Fig. 4), patterns of starch accumulation varied considerably between genotypes and were not obviously correlated with either starch content or final grain weight. For example, two elite cultivars with high grain weights and starch contents in 2013 had very different patterns of starch accumulation during grain filling (Supplementary Text S1). Overall, the data implied that the extent of starch accumulation was determined by the physical limits on grain volume set by developmental processes, rather than grain size and weight being determined by the extent of starch accumulation.

Our data support the notion that final grain weight may be determined primarily by developmental factors such as cell size in either the outer, maternal tissues or in the endosperm. This idea is consistent with evidence that early developmental processes can be important in determining final grain weight. For example, it has previously been shown that the E3 ubiquitin ligase GW2, a factor controlling grain weight that is conserved in species as diverse as Arabidopsis (in which it is named DA1) and rice (Li *et al.*, 2010; Xia *et al.*, 2013), influences carpel growth prior to anthesis in wheat (Simmonds *et al.*, 2016). Other studies have confirmed the importance of carpel size in determining the size of the mature grain (e.g. Calderini *et al.*, 1999, 2001; Xie *et al.*, 2015), although carpel/ovary volume may be influenced by environmental as well as developmental factors (Calderini *et al.*, 1999; Simmonds *et al.*, 2016; Benincasa *et al.*, 2017). Second, a QTL responsible for 7% variation in grain weight has been shown to act by altering the length of pericarp cells, which in turn affects the maximum length to which the grain can grow (Brinton *et al.*, 2017).

Based on the above conclusions, we suggest that in order to achieve higher individual grain weights, breeders and biotechnologists must find ways to increase the cell size and/or number in maternal and/or endosperm tissues of the grain. Higher individual grain weights are unlikely to be achieved by manipulation of either photosynthesis (hence sucrose supply) or starch-synthesising enzymes during grain filling. We emphasise that these conclusions apply to the main period of grain filling. As discussed above, the capacity for starch synthesis and the supply of substrates from photosynthesis may be crucial determinants of various yield parameters in the period around anthesis and early in grain development (see also Tuncel and Okita, 2013). The avoidance of starvation—especially during adverse conditions—and the early establishment of sink strength may influence ovary size (see above) and how many grains will fill (Ji *et al.*, 2010; Guo *et al.*, 2016), and may also impact on individual grain weight at maturity by influencing critical cell division and other developmental processes immediately post anthesis.

Although our results were obtained with winter wheat grown under near-optimal conditions for high yield, our conclusions may be more widely applicable. Many experiments in which assimilate supply to the developing grain has been manipulated—through reduction of photosynthesis (defoliation or shading) or through removal of grains—have led to the conclusion that the supply of assimilates from photosynthesis does not limit the growth of grains. The failure of grain weight to respond to either a limitation or an increase in assimilate supply has been observed in both near-optimal and stress conditions (Acreche and Slafer, 2005; Reynolds *et al.*, 2009, 2005; Serrago *et al.*, 2013).

Despite fact that we did not use replicated plots in our experiments, the application of PCo analysis to provide information about degrees of similarity between genotypes was not compromised because it works on the ‘means’ of the pseudo-replicate data per genotype. The input matrix to the PCo procedure thus contains independent observations, allowing valid comparison of the genotypes. We emphasise that the analysis is descriptive rather than based on formal statistical testing. We also cannot discount the possibility that environment as well as genotype influenced the outcomes of these analyses. Nonetheless this dataset allows the generation of hypotheses to be tested in future work.

Principal coordinates analysis tended to separate the elite cultivars from the landraces when genotypes were grown in the same location in 2014, indicating that these groups may be distinct when multiple parameters are considered together. Dendrograms derived from similarity matrices suggested that the elite cultivars were as diverse as the landraces with respect to most of the variables we measured, even though the range of final grain weights was far greater for landraces than for elite cultivars. This finding appears to run counter to the common belief that plant breeding has seriously reduced genetic diversity in modern, elite varieties (Tanksley and McCouch, 1997). It may also reflect the fact that the variables we measured have not been subject to strong selection during breeding in the recent past. Increased grain number per unit area is a major factor in yield improvement, with a relatively small contribution from variables related to grain filling and grain size (Sadras and Slafer, 2012; Slafer *et al.*, 2014).

## Supplementary data

Supplementary data are available at *JXB* online.

Fig S1. Final grain weights in 2013 and 2014.

Fig S2. Comparison of starch contents at 44 DAA and maturity for genotypes grown in 2014.

Fig S3. Relationship between final grain weight and enzyme activities.

Fig S4. Two-dimensional plots of Principal Coordinates.

Table S1. Full datasets for 2013 and 2014.

Table S2. Pearson correlation coefficients and probability values for the data in Supplementary Table S1.

Table S3. Details of Principal Coordinates.

Table S4. Pedigrees, grain-quality (nabim) classification, and Recommended List data for elite cultivars.

Text S1. Evaluation of two-dimensional plots of Principal Coordinates.

Supplementary Table S1

Supplementary Table S2

Supplementary Tables and Figures
