# Corrigendum to: Final grain weight is not limited by the activity of key starchsynthesising enzymes during grain filling in wheat

**DOI:** 10.1093/jxb/ery372

**Published:** 2018-11-13

**Authors:** Brendan Fahy, Hamad Siddiqui, Laure C David, Stephen J Powers, Philippa Borrill, Cristobal Uauy, Alison M Smith

**Affiliations:** 1 John Innes Centre, Norwich Research Park, Norwich, UK; 2 Rothamsted Research, West Common, Harpenden, Hertfordshire, UK

*Journal of Experimental Botany*, Advance Access Publication: 25 August 2018, doi:10.1093/jxb/ery314

The original online version of this article contained an error in the labelling of graph C (Figure 1). The graph axes were erroneously labelled as measured in (nmol min^-1^ g^-1^ FW). This error has now been corrected.

