# Supplementary material for: Final grain weight is not limited by the activity of key starch-synthesising enzymes during grain filling in wheat
Source: J Exp Bot. 2018 Aug 25;69(22):5461–75. doi: 10.1093/jxb/ery314 (PMC6255701; doi:10.1093/jxb/ery314)
Supplement: Supplementary Tables and Figures [file ery314_suppl_supplementary_tables_and_figures.pdf]

Final grain weight in wheat is not strongly influenced by sugar levels or activities of key starch synthesising enzymes during grain filling

Brendan Fahy, Hamad Siddiqui, Laure C. David, Stephen J. Powers, Philippa Borrill, Cristobal Uauy, Alison M. Smith

Supplementary Material

Supplementary Figure S1. Final grain weights in 2013 and 2014.

Supplementary Figure S2. Comparison of starch contents at 44 DAA and maturity for genotypes grown in 2014.

Supplementary Figure S3. Relationship between final grain weight and enzyme activities.

Supplementary Figure S4. Two-dimensional plots of Principal Coordinates (PCo).

Supplementary Table S3. Principal Coordinates.

Supplementary Table S4. Pedigrees, nabim classification and Recommended List data for elite cultivars.

Supplementary Text 1. Evaluation of two-dimensional plots of Principal Coordinates.

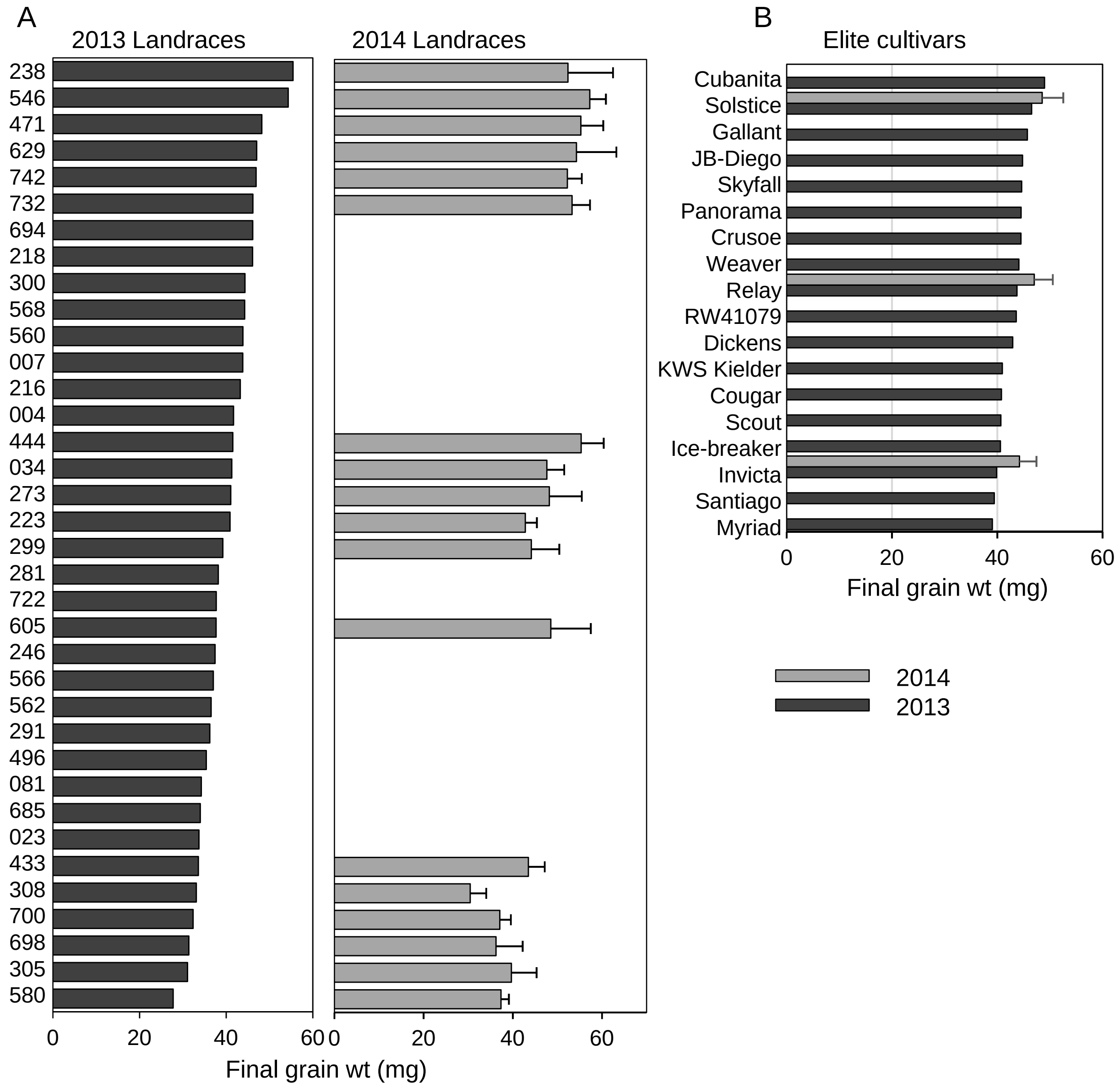

Figure S1. Final grain weights in 2013 and 2014. Values were obtained from thousand-grain weights measured with a Marvin seed analyser. Values for 2013 are means of three technical replicates taken from bulked grain. Values for 2014 are means ± SD of measurements on six samples, each from a separate plant, consisting of grain from the central region of a single ear. A, Landraces. Nomenclature is abbreviated by removal of the initial 1190 common to all genotypes. B, Elite cultivars grown in 2013 (dark grey bars) and 2014 (light grey bars).

Figure S2. Comparison of starch contents at 44 DAA and maturity for genotypes grown in 2014. A, Starch content per mg dry weight at 44 DAA (light grey bars) and per mg flour at maturity (dark grey bars). Values were derived using starch content as % fresh weight (Figure 4A) and the independently-measured dry weight per g fresh weight (Figure 2) at this time point. B, Starch content per grain at 44 DAA (light grey bars) and maturity (dark grey bars). Values are means ± SD of measurements on six samples, each from a separate plant, consisting of grain from the central region of a single ear

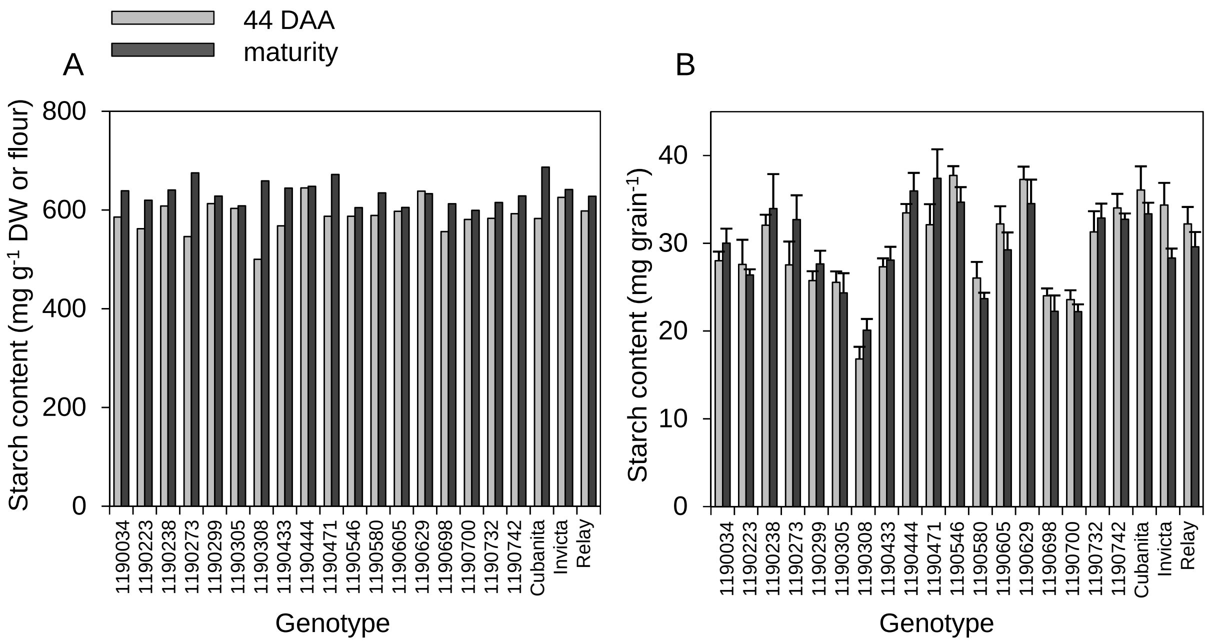

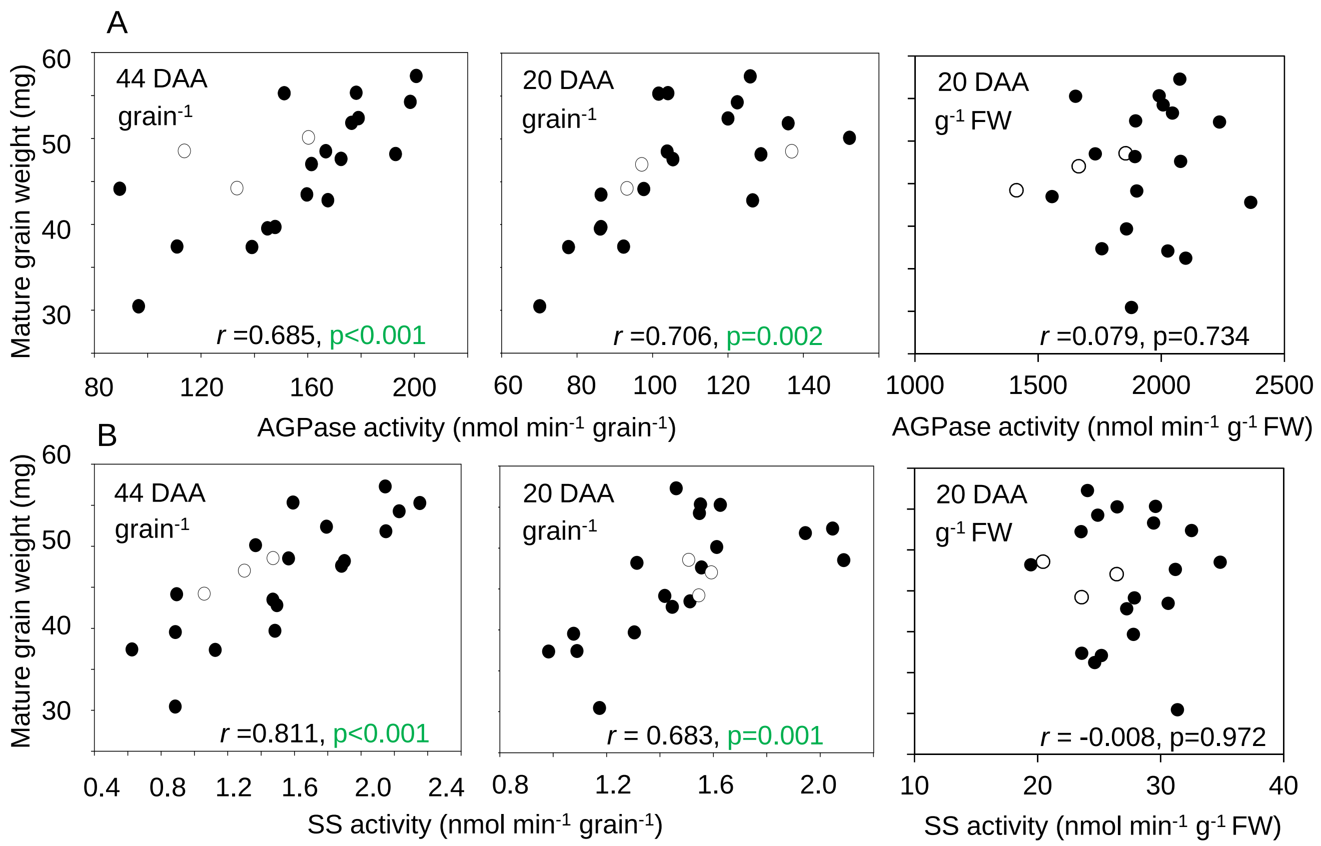

Figure S3. Relationship between final grain weight and enzyme activities. A subset of the data is plotted, complementary to data in Fig. 6. Values are means taken from Supplementary Table S1, which also provides full datasets with genotype designations and measures of variance. The same values are also displayed in different contexts in Fig. 3 (enzyme activities), Fig. S1 and Fig. S2 (mature grain weight and starch content respectively). Black symbols are landraces; white symbols are elite cultivars. *r* is Pearson's correlation coefficient, with n = 21;.p values (F test) are in green where $p<0.05$ (see Supplementary Table S2). A, Correlation between mature grain weight and AGPase activity on a per grain basis at 44 and 20 DAA and on a FW basis for 20 DAA. B, As for A but for starch synthase activity.

Figure S4. Two-dimensional plots of Principal Coordinates (PCo). Plots of the first three PCo are from similarity matrices constructed for the 21 genotypes used in 2014 and the 21 elite cultivars and 36 landraces used in 2013. Euclidean distance measures of similarity were used to reduce the 21x68 data matrix from 2014 and the 21x44 (elite cultivars) and 36x44 (landraces) data matrices for 2013 to 21x21 and 36x36 similarity matrices (Supplementary Table S3), to which PCo analysis was applied. For landraces, nomenclature is abbreviated by removal of the initial 1190 common to all genotypes. A, Elite cultivars grown in 2013. PCo1, 2 and 3 account for 27.88%. 23.25% and 11.06% of the variation in the distance matrix, respectively. B, Landraces grown in 2013. PCo1, 2 and 3 account for 30.97%. 18.72% and 13.99% of the variation in the distance matrix, respectively. C, Genotypes grown in 2014. PCo1, 2 and 3 account for 33.04%. 14.12% and 9.16% of the variation in the distance matrix, respectively.

A

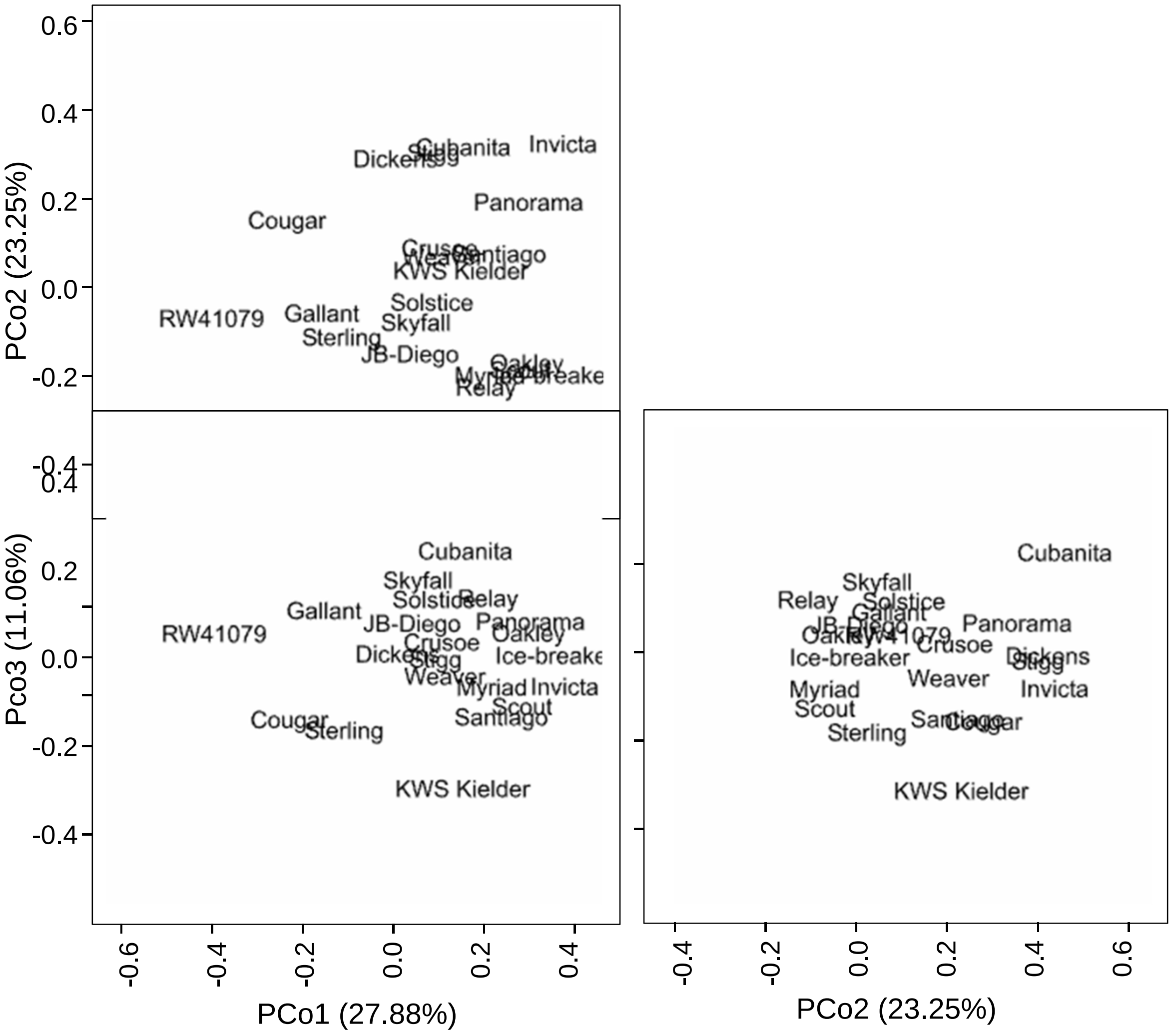

B

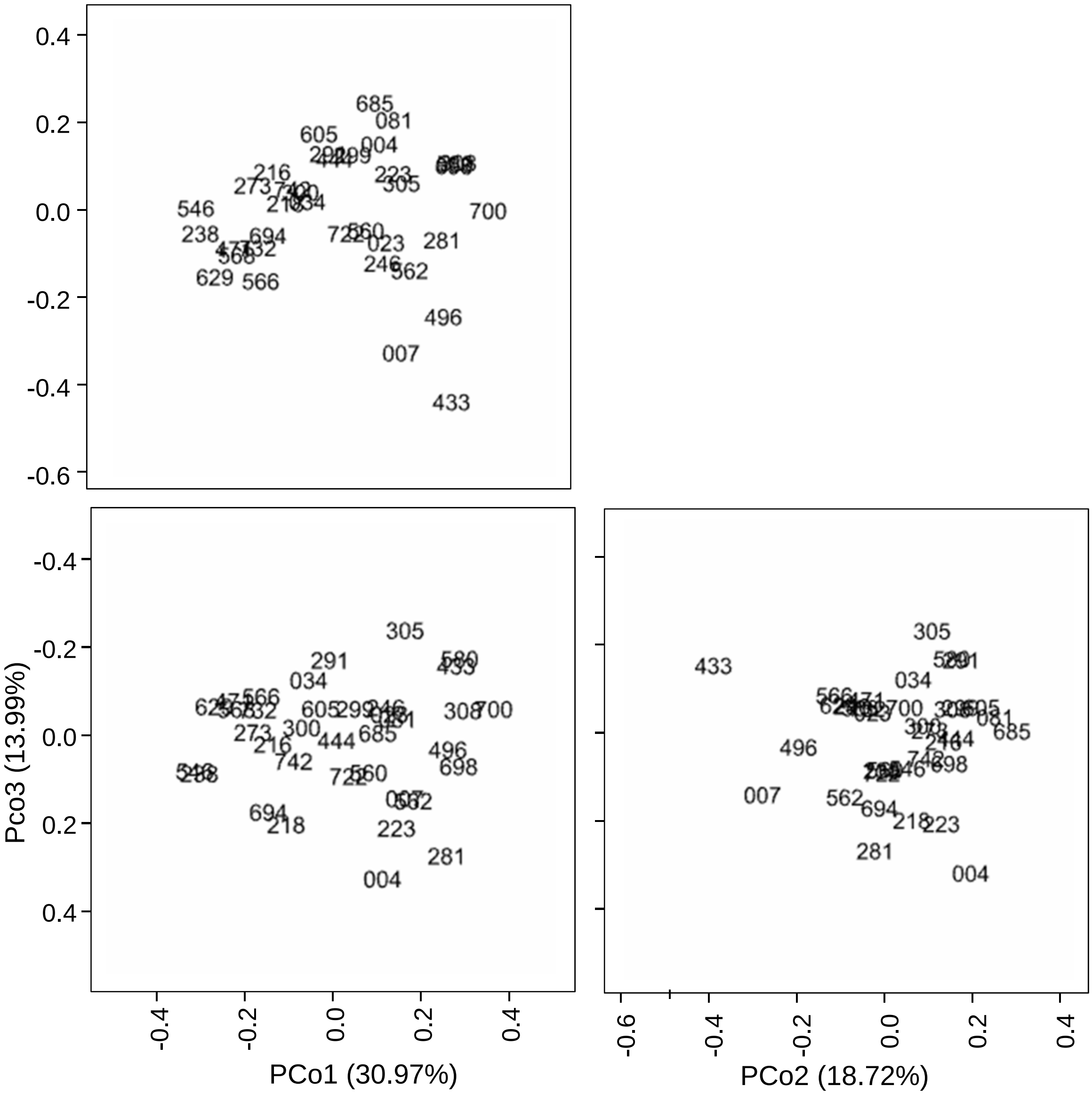

C

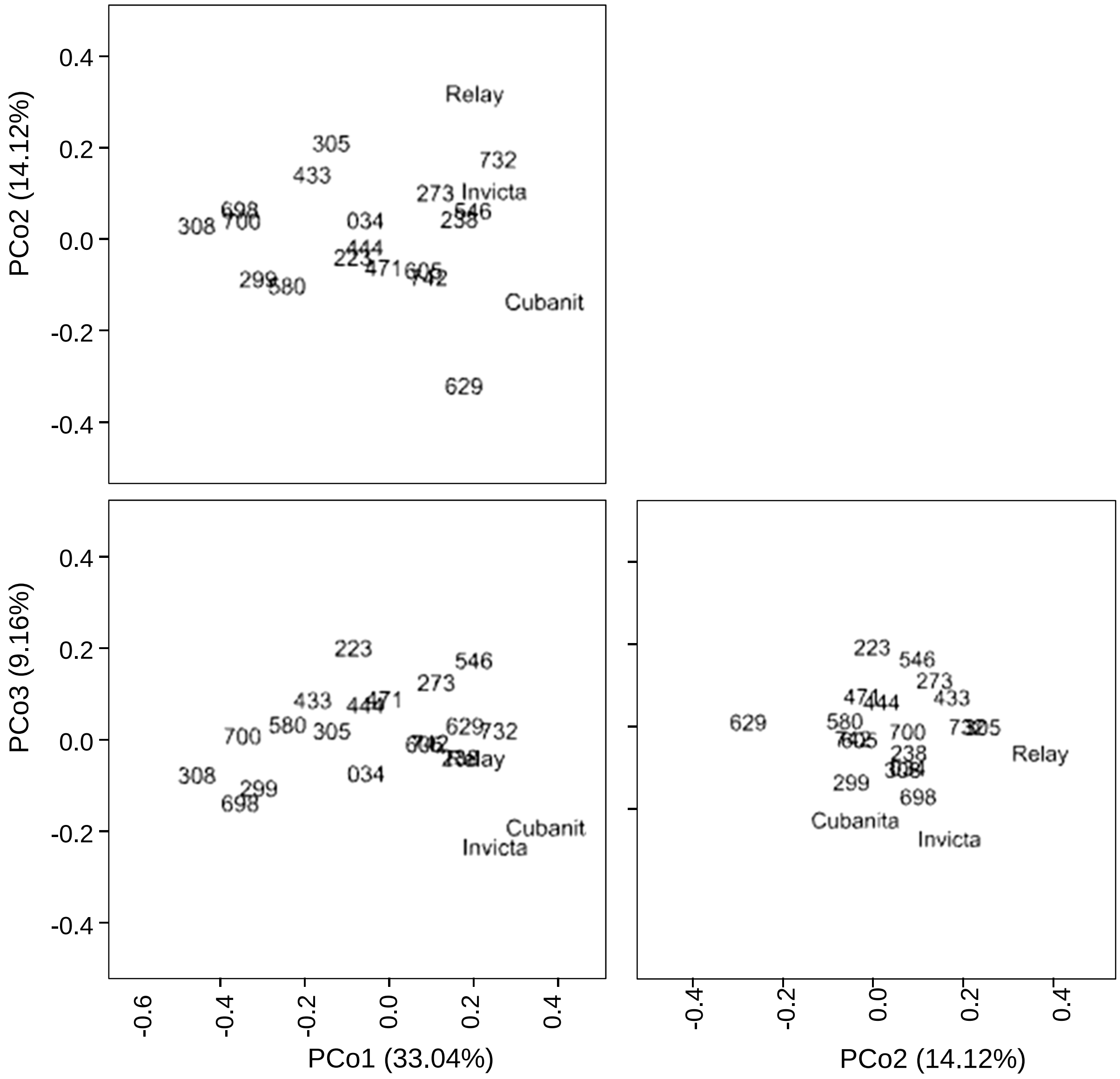

PCo2 (14.12%)
PCo3 (9.16%)
PCo1 (33.04%)
PCo2 (14.12%)
0.4
0.2
0.0
-0.2
-0.4
-0.6
Relay
Invicta
Cubanit
Cubanita
732
305
433
273
546
238
698
700
308
034
444
223
471
605
742
299
580
629

Text S1.

Observations from plots of pairs of Principal Coordinates

In plots of pairs of PCo based on elite cultivars grown in 2013 (Fig. S4A), cultivar Cubanita was peripheral in the PCo2 and 3 dimensions, RW41079 in the PCo1 dimension, and KWS Kielder in the PCo3 dimension. Cubanita and RW41079 had the highest starch contents on a weight basis at maturity, and Cubanita had the highest grain weight. However, the two cultivars differed profoundly during development. RW41079 grains grew very rapidly in the early stages (they had the highest starch content, grain weight and AGPase activity per FW at 10 DAA) whereas Cubitana grains had unexceptional enzyme activity and metabolite contents during the first 30 DAA (Supplementary Table S1). By contrast KWS Kielder had a relatively low final grain weight, and low or unexceptional grain weight, enzyme activity and metabolite content during development (Supplementary Table S1).

For landraces grown in 2013, PCo1 accounted for 31% of the variation in the distance matrix and final grain weight appeared to be important in separating genotypes in this dimension. In plots of pairs of PCo (Fig. S4B), genotypes with high final grain weight (e.g. 546, 238, 629) were at the periphery on the left, and several with low final grain weight were at the periphery on the right (e.g. 700, 698, 580).

In plots of pairs of PCo for the mixture of genotypes grown in 2014 (Fig. S3C), PCo3 particularly distinguished the elite cultivars Cubanita and Invicta from the landraces. Cluster analysis (Fig. 8C) also distinguished Cubanita and Invicta from Relay and the landraces. The landrace 629 was an outlier in the cluster analysis: it had high final grain weight and generally high starch contents and enzyme activity in developing grain, on a FW basis. This genotype also had one of the highest final grain weights and the highest starch content at 30 DAA in the 2013 harvest.

Table S3. Principal Coordinates.

Data are the percentage of variation accounted for by each principal coordinate, and the Latent vector values for PCo 1, 2 and 3 for each genotype. For landraces, nomenclature is abbreviated by removal of the initial 1190 common to all genotypes. Data are plotted in two dimensions in Figure S3.

**Elite cultivars grown in 2013**			
	Percentage variation		
	PCo1	PCo 2	PCo3
	27.88	23.25	11.06
Cultivar	Latent vectors (coordinates)		
	PCo1	PCo2	PCo3
Cougar	-0.3255	0.1299	-0.1397
Crusoe	0.0098	0.0689	0.0302
Cubanita	0.0414	0.2893	0.2302
Dickens	-0.0953	0.2638	0.0042
Gallant	-0.2466	-0.0734	0.0993
Ice-breaker	0.2108	-0.2082	0.0012
Invicta	0.2881	0.2963	-0.0675
JB-Diego	-0.0785	-0.1621	0.0715
KWS Kielder	-0.0081	0.0194	-0.2913
Myriad	0.1254	-0.2092	-0.0686
Oakley	0.2027	-0.1820	0.0500
Panorama	0.1675	0.1691	0.0758
Relay	0.1281	-0.2346	0.1267
RW41079	-0.5201	-0.0847	0.0484
Santiago	0.1213	0.0564	-0.1341
Scout	0.2030	-0.1973	-0.1109
Skyfall	-0.0352	-0.0933	0.1660
Solstice	-0.0145	-0.0494	0.1238
Sterling	-0.2073	-0.1255	-0.1632
Stigg	0.0218	0.2768	-0.0073
Weaver	0.0112	0.0497	-0.0447

**Landraces grown in 2013**			
	Percentage variation		
	PCo1	PCo 2	PCo3
	30.97	18.72	13.99
Landrace	Latent vectors (coordinates)		
	PCo1	PCo2	PCo3
007	0.1341	-0.3198	-0.1311
023	0.0997	-0.0657	0.0582
034	-0.0812	0.0290	0.1349

081	0.1180	0.2174	0.0477
216	-0.1626	0.0978	-0.0094
218	-0.1322	0.0247	-0.1901
223	0.1159	0.0931	-0.1981
238	-0.3272	-0.0445	-0.0751
246	0.0915	-0.1132	0.073
273	-0.2071	0.0665	0.0175
281	0.2287	-0.0597	-0.2602
291	-0.0337	0.1391	0.1799
299	0.0227	0.1371	0.0700
300	-0.0974	0.0504	0.0280
305	0.1349	0.0715	0.2469
308	0.2650	0.1188	0.0664
433	0.2500	-0.4328	0.1670
444	-0.0182	0.127	-0.0008
471	-0.2498	-0.0798	0.0878
496	0.2314	-0.2363	-0.0213
546	-0.3378	0.0141	-0.0699
560	0.0526	-0.0376	-0.0731
562	0.1536	-0.1298	-0.1366
566	-0.1889	-0.1540	0.0979
568	-0.2439	-0.0945	0.0695
580	0.2573	0.1171	0.1826
605	-0.0547	0.1853	0.0706
629	-0.2941	-0.1446	0.0755
685	0.0738	0.2562	0.0158
694	-0.1716	-0.0492	-0.1619
698	0.2557	0.1114	-0.0591
700	0.3344	0.0082	0.0700
722	0.0080	-0.0447	-0.0812
732	-0.1957	-0.0776	0.0680
742	-0.1157	0.0576	-0.0475

**Genotypes grown in 2014**			
	Percentage variation		
	PCo1	PCo2	PCo3
	33.04	14.12	9.19
Landraces and cultivars	Latent vectors (coordinates)		
	PCo1	PCo2	PCo3
034	-0.0329	0.0224	-0.0738
223	-0.0625	-0.0615	0.2105
238	0.1793	0.0243	-0.0378
273	0.1251	0.0844	0.1318
299	-0.2769	-0.1109	-0.1071
305	-0.1114	0.1971	0.0227
308	-0.4165	0.0101	-0.0773
433	-0.1542	0.1256	0.0922
444	-0.0347	-0.0388	0.0813
471	0.0085	-0.0848	0.0951
546	0.2100	0.0442	0.1826

580	-0.2115	-0.1259	0.0366
605	0.0983	-0.0914	-0.0074
629	0.1899	-0.3522	0.0342
698	-0.3186	0.0464	-0.1409
700	-0.3134	0.0211	0.0116
732	0.2669	0.1603	0.0230
742	0.1105	-0.1067	-0.0041
Cubanita	0.3259	-0.1614	-0.1954
Invicta	0.2269	0.0884	-0.2393
Relay	0.1911	0.3093	-0.0384

Year/years on list	Rialto	Robigus	Claire	Cadenza	nabim classification	Pedigree	Cultivar
2014/3		X			Soft Group 4	TUSCAN x ROBIGUS	Cougar
2011/4				X	Group 2	(QUEST x WIZARD) x CORDIALE	Sterling
2013/6				X	Group 1	CORDIALE x GULLIVER	Crusoe
2005/13	X				Group 2	RIALTO x VIVANT	Solstice
2010/9	X			X	Group 1	(CHARGER x MALACCA) x XI19	Gallant
2015/4					Group 1	C4148 x SHD2535	Skyfall
2009/10					Hard Group 4	3351B2 x STRU2374	JB Diego
2008/5		X		X	Hard Group 4	(AARDVARK x ROBIGUS) x ACCESS	Oakley
2010/8		X			Group 3	Z435 x DEBEN	Scout
2014/5			X		Soft Group 4	NSL00-0742 x NIJINSKY	Myriad
2013/6					Hard Group 4	GLADIATOR x VECTOR	Relay
2015/2	X			X	Group 2	CORDIALE x KETCHUM	Cubanita
2010/7	X			X	Group 2	(SOLSTICE x XI19) x SOLSTICE	Panorama
2011/6		X	X		Group 3	NSLWW48 x ROBIGUS	Invicta
2014/5					Hard Group 4	DEFENDER x W01SEC322	Dickens
2012/7		X		X	Hard Group 4	OAKLEY x SHERBORNE	Santiago
2012/2					Hard Group 4	(BISCAY x LW96-2930) x TANKER	Stigg
2014/4				X	Hard Group 4	BROMPTON x OAKLEY	KWS Kielder

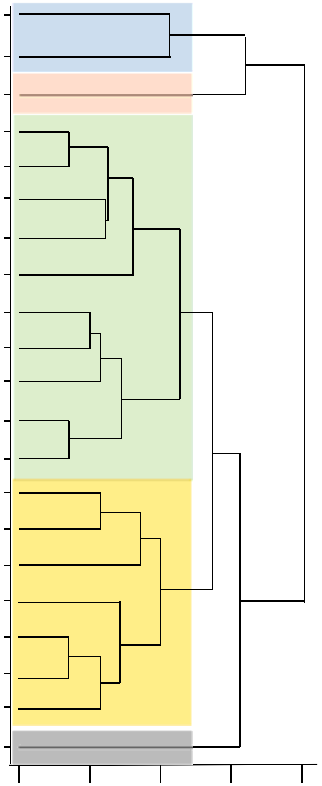

Table S4. Pedigrees, nabim classification and Recommended List data for elite cultivars. The dendrogram (right) is from Fig. 8A, and represents relationships derived from similarity matrices for elite cultivars grown in 2013. The colored blocks show the four clusters apparent at a similarity score of 0.86. The Table shows the pedigrees of 18 of the cultivars. Contributions of four parents shared by several cultivars are indicated in separate columns, and by use of the same color-coding in the pedigree column. Classification of cultivars by grain quality and uses is from the UK flour millers organisation nabim (http://www.nabim.org.uk/wheat/wheat-varieties). The Year/years on list column shows the year in which the cultivar was introduced to the UK Recommended List [Agriculture and Horticulture Development Board (AHDB); https://cereals.ahdb.org.uk/varieties/ahdb-recommended-lists.aspx], and the number of years it remained on the list. Dark colors indicate short duration (2-3 years); progressively lighter colors indicate progressively longer periods on the list. Note the lack of correspondence between the clusters in the dendrogram and the pedigree of the cultivars, their nabim classification, and their year of introduction/duration on the Recommended List.
